# Artificial intelligence in cardio-oncology: decoding mechanisms, predicting toxicity, and personalizing cancer therapy

**DOI:** 10.3389/fcvm.2026.1761811

**Published:** 2026-04-29

**Authors:** Chengqi Yu, Leilei Jiang, Liuhua Long, Huiming Yu

**Affiliations:** 1 National Cancer Center/National Clinical Research Center for Cancer/Cancer Hospital, Chinese Academy of Medical Sciences and Peking Union Medical College, Beijing, China; 2 Key Laboratory of Carcinogenesis and Translational Research (Ministry of Education/Beijing), Department of Radiation Oncology, Peking University Cancer Hospital & Institute, Beijing, China

**Keywords:** artificial intelligence, cancer survivorship, cardio-oncology, cardiotoxicity, precision medicine, risk prediction

## Abstract

Cancer therapy-related cardiovascular toxicity (CTR-CVT) threatens the sustainability of oncological advancements, demanding innovative approaches for early risk stratification. This review synthesizes how artificial intelligence (AI) is redefining cardio-oncology through multimodal integration of multi-omics, dynamic imaging, and real-world biosensor data. By decoding novel pathophysiological mechanisms and enabling continuous risk reclassification, AI transcends traditional static paradigms to generate patient-specific toxicity trajectories. Crucially, AI-driven interventions shift clinical practice from reactive monitoring to preemptive cardioprotection. While challenges in data heterogeneity, model interpretability, and equitable implementation persist, emerging solutions like federated learning and explainable AI pave the way for robust clinical translation. We hope that this review will summarize the current state of emerging applications of machine learning and AI in precision medicine predictive modeling, providing direction for AI-enabled precision cardiovascular oncology—ensuring the effectiveness of cancer treatment while safeguarding long-term cardiovascular health through personalized risk mitigation measures.

## 1 Introduction

Advances in cancer therapeutics have revealed a growing burden of treatment-related cardiotoxicity. Chemotherapy drugs, targeted agents and immune checkpoint inhibitors (ICIs) confer distinct yet significant risks of cardiomyopathy, arrhythmias, and vasculopathy, compromising long-term survival and limiting therapeutic efficacy. Current risk stratification paradigms, such as the European Association of Cardiovascular Imaging/Heart Failure Association (EACVI/HFA) cardio-oncology protocol, rely heavily on static clinical variables and periodic imaging. These approaches suffer critical limitations: inadequate sensitivity for early subclinical injury, insufficient integration of dynamic biomarkers, and resource-intensive monitoring that strains healthcare systems. Consequently, more effective strategies to predict and mitigate cardiotoxicity are urgently needed.

Artificial intelligence (AI)—particularly machine learning (ML) and deep learning (DL)—offers transformative potential to overcome these barriers (1–5). AI assistance can better understand patients’ test results, notice details that are invisible to the human eye, and thereby assist doctors in making judgments (2–4). By harmonizing multimodal data streams, including electronic health records (EHRs), cardiac imaging (echocardiography, cardiovascular magnetic resonance), genomics, proteomics, and real-time biosensor data, AI can generate dynamic, patient-specific toxicity trajectories. This capability transcends traditional risk scores, enabling continuous predictive refinement throughout the cancer journey. Moreover, AI-driven analysis of high-dimensional data provides unprecedented opportunities to decode complex pathophysiological mechanisms linking oncotherapies to cardiovascular injury, revealing novel therapeutic targets.

**Figure 1:**
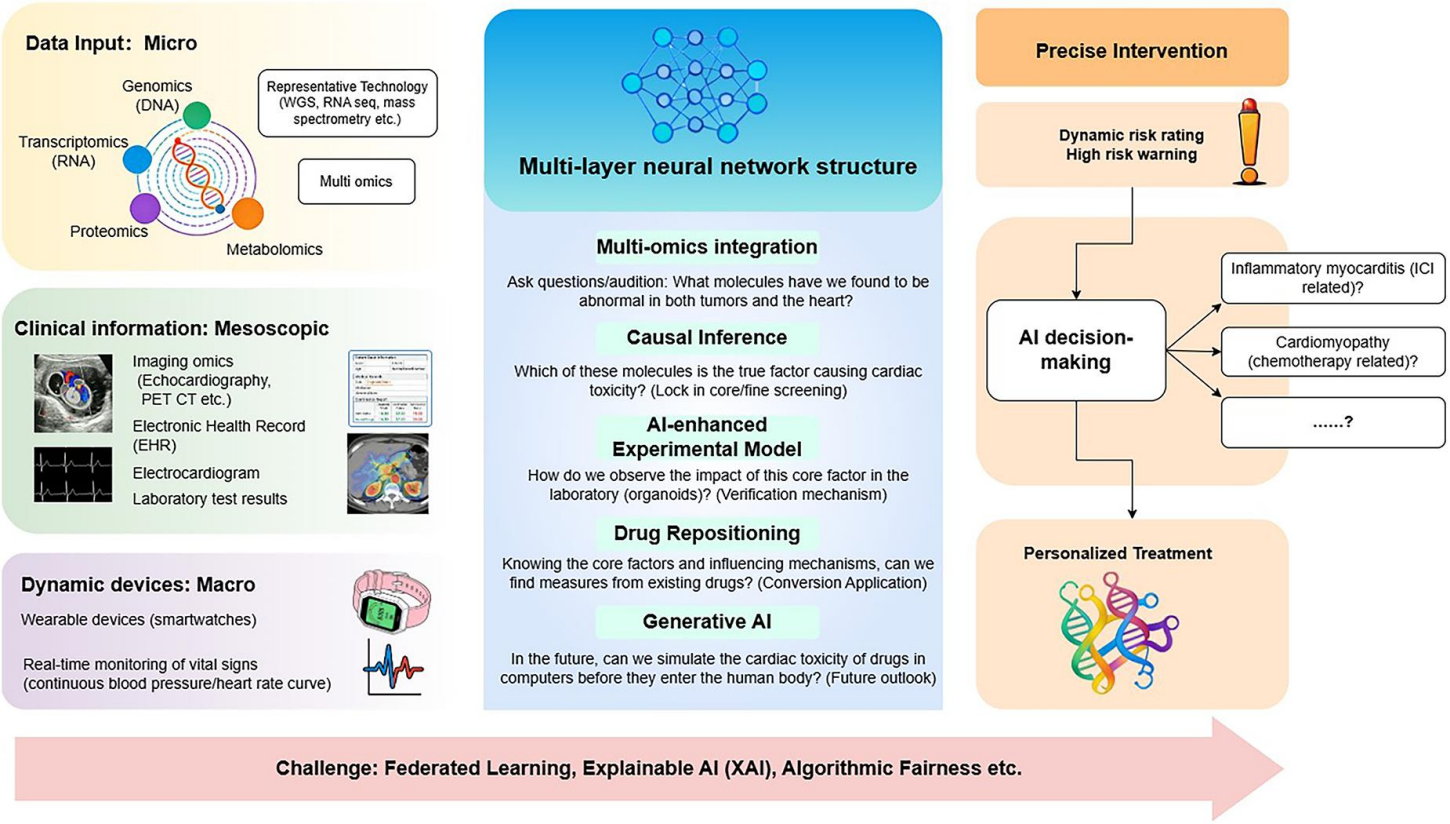
AI-Mediated workflow from multi-omics to clinical phenotypes.

This review synthesizes emerging applications of AI in cardio-oncology, focusing on three pillars: (1) mechanistic decoding of cardiotoxicity pathways via multi-omics integration; (2) dynamic prediction models for early toxicity detection and risk reclassification; and (3) personalized therapeutic optimization through computational decision support. Finally, we critically examined the challenges of transformation and proposed future prospects, hoping that this article will systematically summarize and emphasize the key role of artificial intelligence in advancing precision medicine in the field of cardiac oncology (Figure 1).

## 2 AI-Driven Mechanistic Insights into Cardiotoxicity

The molecular cascades underlying cancer therapy-induced cardiotoxicity—from anthracycline-triggered DNA damage to immune checkpoint inhibitor-associated myocarditis—remain a critical frontier in biomedical research. Traditional mechanistic studies, often reliant on labor-intensive models with limited human translatability, are increasingly being complemented by artificial intelligence (AI) approaches. By integrating multi-omics, electronic health records, and imaging data, AI enables a systems-level dissection of cardiotoxicity, uncovering latent, nonlinear relationships that conventional methods may overlook.

We focus on five interconnected domains where AI is advancing mechanistic discovery, progressing from foundational insights to translational applications. First, multi-omics integration identifies shared pathogenic pathways and molecular networks common to both cancer and cardiovascular disease. Building on these associations, causal inference models then distinguish correlational findings from true causal drivers. These hypotheses are further tested and refined using AI-enhanced experimental platforms—such as organoids and high-content imaging—that recapitulate inter-organ communication and functional phenotypes. The resulting mechanistic understanding informs AI-driven drug repurposing frameworks, enabling systematic identification of dual-purpose agents. Finally, generative AI and structural modeling offer a forward-looking approach to simulate molecular interactions and predict potential cardiotoxic effects prior to clinical exposure.

### 2.1 Multi-omics integration uncovers shared molecular mechanisms in cardio-oncology

AI-powered integration of multi-omics data enables a systems-level understanding of the molecular crosstalk between cancer and cardiovascular disease (1, 2). By systematically analyzing large-scale proteomic, genomic, and transcriptomic datasets, these approaches have revealed shared inflammatory and immune-regulatory pathways that underlie cardio-oncological comorbidities and therapy-related toxicities (3–5).

At the proteome-wide level, The predictive effect of proteomics on cardiac tumor comorbidity has been confirmed (6). A comprehensive proteomic phenomenon map was constructed using AI models such as machine learning, covering 2920 plasma proteins and 1706 diseases from 53,026 individuals, indicating that at least 50 diseases share over 650 proteins (7). Notably, TNF family proteins such as EDA2R showed significant enrichment across both lymphomas and circulatory diseases, while GDF15 emerged as a master predictor for over 200 conditions, underscoring systemic inflammation as a common thread linking cancer and cardiovascular pathology (7, 8). These findings align with systematic mechanistic studies elucidating how TNF superfamily molecules—acting as key co-stimulatory immune checkpoints—regulate lipid metabolism, plaque evolution, and thrombosis through conserved signaling cascades (9).

At the genomic level, genome-wide pleiotropy analysis identified 60 shared susceptibility loci between coronary artery disease and four common cancers, with 35 loci exhibiting opposing effects on the two conditions (10). Integration with transcriptomic and proteomic data revealed that 13 of these loci influence disease risk through differential gene expression, including CALCRL and ANGPTL4—targets of approved or investigational drugs—highlighting potential repurposing opportunities (10).

At the transcriptomic level, machine learning algorithms applied to cross-disease datasets have identified shared gene signatures. Studies combining differential expression analysis with LASSO and random forest identified ADD3 and ATP2B4 as common key genes in prostate cancer and coronary heart disease, both enriched in immune-related pathways and correlated with immune cell infiltration (11). Similarly, integration of transcriptomics with Mendelian randomization in myocardial infarction and gastric cancer revealed 74 common differentially expressed genes enriched in ubiquitin-dependent proteasome pathways, with eight diagnostic genes achieving excellent predictive performance (AUC > 0.95) (12).

Collectively, these multi-omics approaches—spanning proteomics, genomics, and transcriptomics—demonstrate that inflammation, immune dysregulation, and metabolic disturbances serve as shared molecular foundations linking cancer and cardiovascular disease, providing a unified framework for understanding comorbidity and therapy-induced cardiotoxicity. AI can infer and respond to complex patterns in data, so many expert consensus and guidelines (1, 13) recommend using machine learning for causal inference analysis to understand the mutual causal effects between tumor occurrence, development, and cardiovascular disease.

### 2.2 From association to causation – multi-dimensional causal inference models for unraveling shared mechanisms

Traditional biomarker studies have largely relied on association analyses, which often fail to distinguish causal drivers from passive correlates. Recent advances in computational causal inference have enabled a paradigm shift toward identifying true causal factors and pathways underlying cardio-oncology comorbidities.

Formal causal reasoning employing the Halpern-Pearl actual causality model provides a rigorous counterfactual framework to pinpoint molecular events that are causally necessary for disease occurrence. Guha et al. (3) integrated protein corona proteomics with this model and identified thromboxane-A synthase 1 (TBXAS1) as a causal factor linking atherosclerotic cardiovascular disease (ASCVD) and metastatic prostate cancer (mPC). TBXAS1, undetectable in conventional plasma proteomics, emerged as a shared causal driver of platelet aggregation, vascular dysfunction, and tumor progression, illustrating the power of coupling advanced experimental techniques with formal causal logic.

Probabilistic graphical models, such as Bayesian networks, offer an intuitive way to represent causal dependencies while handling real-world data challenges like missingness and rare events. Bernasconi et al. (14) developed the first Bayesian network to predict 5-year cardiovascular disease risk in adolescent and young adult breast cancer survivors. By combining population-based and clinical cohorts, and explicitly modeling selection bias through a context node, the model achieved high predictive accuracy and causal interpretability, enabling clinicians to prioritize high-risk patients for personalized follow-up.

Genetic causal inference leverages the random assortment of germline variants to infer causality free from confounding. Genome-wide pleiotropy and colocalization analyses have identified numerous shared susceptibility loci for coronary artery disease and multiple cancers (10, 15), providing orthogonal evidence for shared pathogenic mechanisms.Causal mediation analysis using Mendelian randomization further dissects the pathways linking genes to diseases via intermediate molecular traits. Carreras-Torres et al. (15) performed a multiomic integration of metabolite GWAS, transcriptome-wide association, and mediation analysis, demonstrating that atherogenic metabolite levels (e.g., LDL cholesterol, triglycerides) mediate the effects of immune-related genes (e.g., HCG27) on cardiovascular risk. This approach provides a blueprint for deciphering gene–metabolite–disease causal chains.

Together, these multi-dimensional causal inference strategies—ranging from formal counterfactual reasoning to probabilistic graphical models, genetic instrumental variable analyses, and mediation frameworks—are transforming our ability to move beyond correlation and uncover the causal architecture underlying cardio-oncology comorbidities (1). This integrated causal perspective not only enhances mechanistic understanding but also guides the development of targeted interventions and personalized risk stratification.

### 2.3 AI-augmented experimental models for mechanistic deconvolution

The complexity of cardio-oncology demands experimental models that transcend traditional approaches. Recent advances have integrated artificial intelligence (AI) with *in vitro* and *ex vivo* platforms—such as iPSC-derived cardiomyocytes, organoids, and microphysiological systems—enabling dynamic, multi-parametric dissection of disease mechanisms (16, 17). Organoids have emerged as particularly valuable tools, recapitulating human tissue complexity for toxicity assessment across multiple organ systems (18).

AI-powered high-content screening accelerates mechanistic discovery. Ma et al. combined miRNA sequencing with machine learning-based target prediction to uncover that exosomal transfer of miR-216a-5p from doxorubicin-treated breast cancer cells to cardiomyocytes exacerbates cardiotoxicity via the ITCH/TXNIP/NLRP3 pathway (19). This approach reveals inter-organ communication that traditional methods might miss.

Deep learning enhances phenotypic analysis across scales. At the single-cell level, deep learning quantifies contractile dynamics of iPSC-CMs from holographic images, achieving high accuracy in classifying drugs by their mode of action (20). In clinical imaging, deep learning-based classification confidence improves echocardiographic quality assessment, reducing false-positive rates in cancer therapy-related cardiac dysfunction monitoring (21). Especially for 3D organoids, deep learning networks automatically segment internal structures from optical coherence tomography, enabling quantitative assessment of beating and chamber formation (22). Generative and multi-task AI further expand capabilities. Conditional generative adversarial networks perform “virtual staining” of cardiac organoids from phase-contrast images (23), while multi-task neural networks predict multiple cardiotoxicity endpoints simultaneously by leveraging shared representations across molecular initiating events (24).

Finally, AI-guided design optimization closes the loop from analysis to engineering. Combining micropatterning with manifold learning identifies calcium transient upstroke time as the key feature distinguishing functional phenotypes, enabling rational design of geometrically confined organoids with enhanced sarcomere alignment (25). Integration with multi-organ chips and AI-powered analysis promises deeper insights into systemic drug effects (18).

In summary, the convergence of AI with advanced experimental models creates a powerful feedback loop: high-content screening uncovers mechanisms, deep learning provides rich phenotypic readouts, generative models augment throughput, and machine learning guides model design—accelerating the translation of preclinical discoveries into safer cardio-oncology therapies.

### 2.4 AI-enhanced drug repurposing for cardioprotection

**Figure 2:**
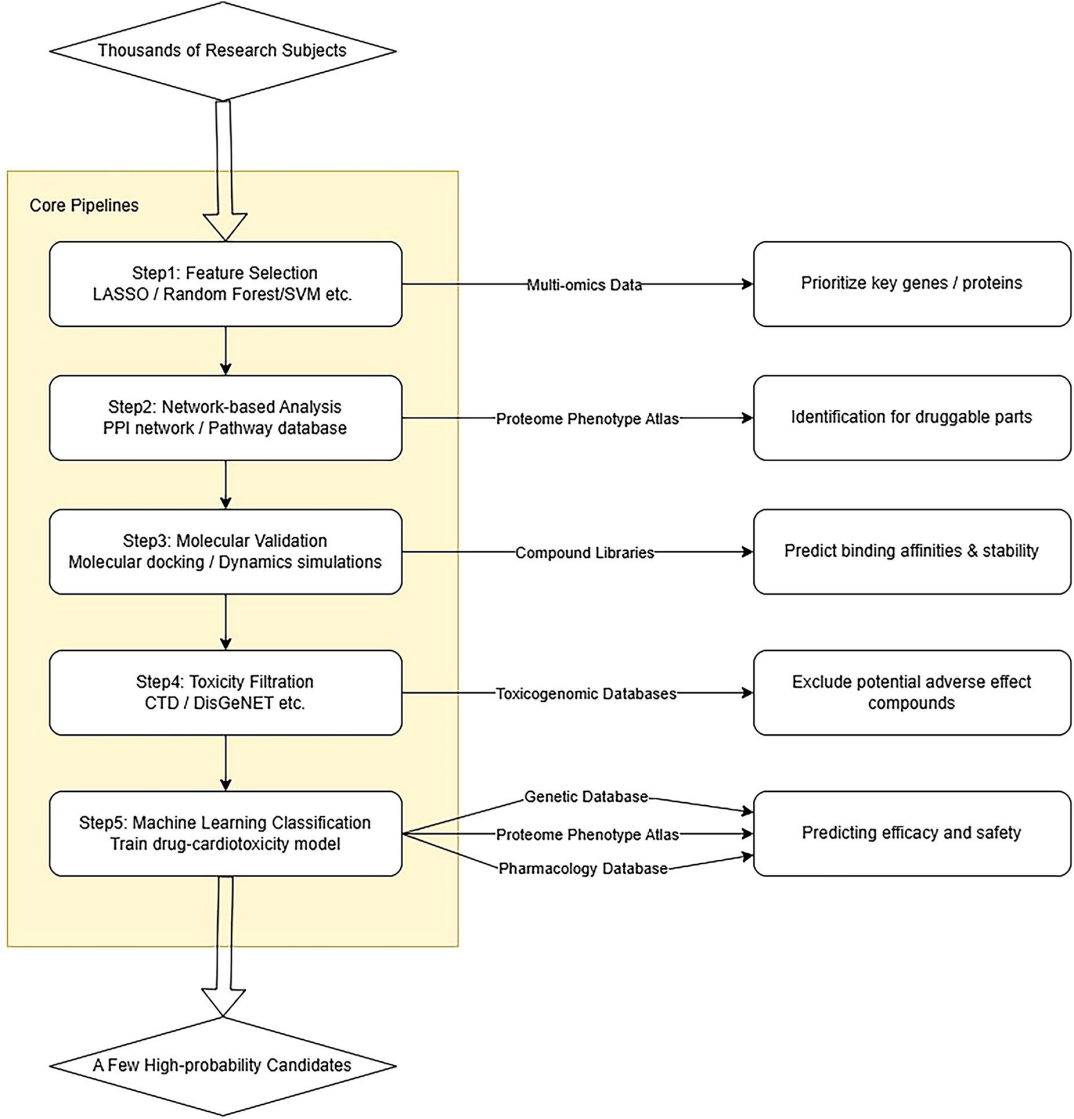
Core mechanism framework for AI recognition of heart protective drugs. In the context of drug repurposing, AI algorithms specifically identify cardioprotective agents through several key mechanisms: (1) feature selection methods (e.g., LASSO regularization, random forest importance scores) prioritize genes or proteins most strongly associated with both disease pathology and potential drug interactions; (2) network-based approaches map these prioritized targets onto protein-protein interaction networks and pathway databases to identify druggable nodes within disease modules; (3) molecular docking and dynamics simulations then computationally screen compound libraries against these prioritized targets to predict binding affinities and stability, with binding energies ≤−5 kcal/mol typically indicating stable interactions; (4) integration of toxicogenomic databases (e.g., CTD, DisGeNET) enables early filtering of compounds with potential adverse effects; (5) machine learning classifiers trained on known drug-cardiotoxicity associations can predict the likelihood of both therapeutic efficacy and cardiac safety for candidate compounds. These computational pipelines systematically narrow the search space from thousands of compounds to a few high-probability candidates for experimental validation.

AI-driven drug repurposing integrates multi-omics data, network toxicology, and machine learning to systematically identify existing compounds with dual antitumor and cardioprotective effects (Figure 2). This approach addresses a critical clinical need: expanding therapeutic options while mitigating cardiotoxicity. Large-scale resources, including comprehensive proteome-phenome atlases and platform-based strategies like the disease-drug correlation method, provide the foundation for such efforts by enabling systematic candidate identification across disease classes (7, 26).

Multi-layered computational pipelines have successfully validated specific candidates through integrated bioinformatics and machine learning (4, 5, 27). These studies typically employ algorithms including LASSO, random forest, and support vector machines to prioritize key genes from transcriptomic data, followed by molecular docking and dynamics simulations to validate binding stability. Emerging technologies are expanding these capabilities: large language models now enable high-accuracy ADME/ADMET prediction (27), while natural language processing of electronic medical records has independently validated cardioprotective effects of common cardiovascular drugs in real-world patient populations (28). Network pharmacology integrated with RNA-seq further elucidates mechanistic pathways underlying cardioprotection (29).

By systematically narrowing the search space from thousands of compounds to high-probability candidates, AI-driven drug repurposing accelerates the discovery of safer cancer therapies and provides mechanistic insights into their cardioprotective effects.

### 2.5 Generative AI and target simulation for cardiotoxicity prediction

Generative artificial intelligence and deep learning have enabled high-resolution modeling of protein structures and their dynamic interactions, providing a powerful framework for predicting the cardiac safety of anticancer therapeutics. AI-driven structure prediction methods, such as AlphaFold and RoseTTAFold, have been successfully applied to model cardiac ion channels (e.g., Nav1.5, Cav1.2) and protein complexes like VANG1/SCRIB/NOS1AP, the latter including NOS1AP—a protein implicated in myocardial repolarization and QT interval regulation (30–32). Molecular dynamics simulations further elucidate drug–target interactions at the atomic level, as demonstrated in studies of anthracycline-induced cardiotoxicity, where drug–membrane interactions were shown to depend on both drug form and membrane complexity (33). These mechanistic insights have been translated into predictive platforms such as hERG-LTN and CUPID, which integrate generative molecular embeddings and explainable AI to assess blockade risk across multiple cardiac ion channels and identify toxic chemical substructures (34, 35). Collectively, these computational approaches offer a mechanistic basis for early identification of cardiotoxicity risks, enabling prioritization of safer drug candidates prior to experimental validation.

## 3 Clinical translation: from prediction to intervention

AI's true promise in cardio-oncology lies in bridging risk assessment with actionable interventions, transforming passive surveillance into dynamic precision care. This evolution begins with AI-enhanced risk stratification. While current guidelines use static categories, ML algorithms synthesize multimodal EHRs—treatment history, genomic susceptibility, and emerging biomarkers—to generate dynamic risk trajectories.

### 3.1 AI-enhanced imaging and hemodynamic monitoring for dynamic risk reclassification

On the one hand, artificial intelligence constructs predictive models that exceed human visual resolution capabilities by mining the spatiotemporal characteristics of echocardiograms, cardiac magnetic resonance imaging (CMR), and biomarkers, thereby improving the accuracy of patient condition assessment. A DenseNet-121-based convolutional neural network (CNN) quantified echocardiographic image quality via classification confidence (CC) in apical four-chamber (A4C) views; when CC >900, left ventricular global longitudinal strain (LVGLS) measurements showed improved agreement with CMR, reducing false-positive cancer therapy-related cardiac dysfunction (CTRCD) diagnoses (21). Such strategies extend single-timepoint LVGLS assessments to continuous monitoring. The EchoNet-Dynamic model integrates ultrasound video sequences through a 3D convolutional neural network to achieve beat-by-beat EF prediction, detecting trastuzumab-induced subclinical dysfunction earlier than physicians while achieving an average absolute error of only 6.0% in predicting EF in an external cohort (36). Similarly, AI-derived coronary artery calcium (AI-CAC) scoring from radiotherapy planning CT scans identified patients at high myocardial infarction risk (score >400), guiding preemptive statin initiation (37).

### 3.2 Multimodal integration for etiological differentiation and therapeutic personalization

On the other hand, AI further advances multidimensional risk modeling by integrating cardiac imaging, functional indices, and clinical data to predict CTRCD risk factors (37), converting imaging archives into proactive defense systems. Continuous monitoring technologies extend vigilance beyond clinics, capturing real-world cardiotoxicity signatures missed by episodic assessments. AI-ECG algorithms decode subtle electrical biomarkers predictive of future injury: CNNs analyzing baseline ECGs detected electrical remodeling—a harbinger of anthracycline- or trastuzumab-induced cardiomyopathy—correlating with increased CTRCD incidence in high- and low-risk cohorts (38). Wearable devices enhance this capability, for example, smartwatch photoplethysmography sensors coupled with recurrent neural networks enable preemptive mobile health interventions. Therefore, AI-driven therapeutic interventions represent a shift from empirical decision-making to mechanistic optimization, transforming passive data streams into preclinical warning systems. Artificial intelligence can not only detect toxicity but also automatically take corrective measures through integrated clinical pathways.

A critical frontier in this translational pathway is the etiological differentiation of myocardial injury—specifically, distinguishing between inflammatory myocarditis (e.g., immune checkpoint inhibitor-associated) and traditional drug-induced cardiomyopathy (e.g., from anthracyclines or trastuzumab). AI-ECG algorithms are uniquely positioned to address this challenge by decoding distinct electrophysiological signatures that precede overt clinical phenotypes. Recent systematic reviews highlight that machine learning models applied to electrocardiography can achieve high diagnostic accuracy in detecting acute myocarditis, leveraging subtle repolarization abnormalities and spatiotemporal QRS complex changes that escape conventional interpretation (39). For instance, advanced neural networks combining improved quantum genetic algorithms with backpropagation have successfully differentiated myocarditis from myocardial infarction by analyzing raw ECG signals and heart rate variability, reaching up to 90% classification accuracy in distinguishing inflammatory from ischemic etiologies (40). Extending this concept to cardio-oncology, multimodal AI frameworks integrating serial ECGs with high-sensitivity troponin kinetics and CMR radiomics could potentially decouple the mechanistic pathways: type I (inflammatory) injury mediated by T-cell infiltration vs. type II (toxic) injury from oxidative stress. By training on biopsy-proven cohorts, deep learning models may learn to recognize the electrical remodeling patterns characteristic of cytokine release and myocardial edema, thereby enabling non-invasive discrimination between these entities. Such capability would have profound therapeutic implications—guiding the use of immunosuppression vs. cardioprotective agents without requiring routine endomyocardial biopsy.

These advances facilitate precision therapy personalization. AI integration of multi-omics data (genomic, proteomic) tailors treatment selection. For example, pathway mutation analysis in tumor cells enables AI-guided recommendations of sensitive targeted agents (e.g., KRAS G12C inhibitors) (41). The proprietary software “ICI-MACE Risk Predictor v1.0” (Copyright Registration No. 9199415, China), developed by Zhongshan Hospital, Fudan University, integrates novel myocardial injury biomarkers to decode the “anti-tumor immunotherapy → biomarker flux → myocardial injury” cascade. Future iterations will establish hospital-to-home monitoring networks for end-to-end management of high-risk patients. These applications of AI technology for precision treatment in the field of oncology and cardiology have entered clinical use, laying the foundation for future scientific research and conversion.

## 4 Bottlenecks and potential solutions of AI translation in cardio-oncology

Despite AI’s compelling potential to decipher cancer therapy-related cardiovascular toxicity (CTR-CVT) mechanisms, predict individualized risk, and optimize cancer treatment, its translation into clinical practice faces substantial barriers.

### 4.1 Data fragmentation and model generalizability

Data fragmentation and limited model generalizability represent primary challenges. Heterogeneity in multicenter data—spanning cancer-specific cardiotoxic phenotypes (e.g., HER2 + breast cancer) to emerging therapies (e.g., CAR-T cell therapy) and variable imaging/omics features—constrains AI generalizability. Small sample sizes for rare but life-threatening events (e.g., CAR-T-associated cardiomyopathy) are exacerbated by restricted data sharing under privacy regulations and institutional silos. Emerging solutions like federated learning (enabling collaborative training without raw data exchange) and generative adversarial networks (synthesizing realistic data) offer promising avenues to overcome these limitations (42).

### 4.2 The “black Box” nature of AI models and the imperative for explainability

The inherent “black box” nature of AI models creates explainability challenges that undermine clinical trust. Clinicians and patients struggle to interpret AI-driven decisions, hindering validation, informed consent, and accountability. Establishing causality rather than mere correlation for CTR-CVT is essential.

While explainable AI (XAI) tools like SHAP provide feature attribution to identify influential variables, emerging counterfactual explanation methods offer a complementary paradigm that addresses “what-if” questions directly relevant to clinical decision-making (43). Unlike SHAP's retrospective attribution, counterfactual explanations prospectively identify the minimal changes in input features required to alter a model's prediction—directly supporting what clinicians intuitively ask: “What would need to be different for this patient to avoid CTR-CVT?” (44). In cardio-oncology contexts, this approach has demonstrated practical utility; for instance, recent work by Balordi et al. employed counterfactual analysis within Pearl's structural causal model framework to evaluate whether ovarian suppression therapy in adolescent and young adult breast cancer survivors is a necessary or sufficient cause for ischemic heart disease, hypertension, and dyslipidemia (45). Their findings revealed that while ovarian suppression is necessary for ischemic heart disease (probability of necessity >97.8%), it is rarely sufficient alone (probability of sufficiency <1.97%), whereas it could induce hypertension in approximately 30% of cases independently—insights directly informing personalized follow-up strategies (45). This counterfactual reasoning transcends conventional correlation-based explanations by quantifying causal probabilities (necessity and sufficiency), enabling clinicians to distinguish core drivers from incidental findings and prioritize monitoring for specific outcomes based on treatment exposure.

Beyond *post hoc* XAI methods, several alternative strategies can enhance model transparency and foster clinical trust. Interpretable models—those designed with intrinsic transparency rather than retrofitted explanations—offer a compelling solution. Models such as generalized linear models (GLMs), decision trees, and rule-based systems provide explicit, human-readable decision logic that clinicians can directly verify without relying on approximation-based explanations. Recent evaluations in healthcare contexts have demonstrated that rule-based approaches like RuleFit and RuleMatrix consistently provide robust and interpretable global explanations across diverse clinical tasks, outperforming *post hoc* methods in both fidelity and stability (46). Although inherently interpretable models may incur a modest performance penalty (typically 5%–7% reduction in AUC compared to black-box alternatives) (47), this trade-off may be clinically acceptable in cardio-oncology applications where understanding the rationale behind a risk prediction is paramount for therapeutic decision-making. Furthermore, XAI-embedded ensembles represent a hybrid paradigm wherein multiple explainable models are combined to achieve competitive predictive performance while preserving transparency. Such ensembles can leverage the complementary strengths of interpretable base learners (e.g., shallow decision trees, rule-based classifiers) to generate predictions accompanied by inherently traceable reasoning chains. Embedding XAI into workflows—demystifying technical logic—is crucial to bridge trust gaps and meet regulatory requirements.

### 4.3 Algorithmic bias and healthcare disparities

Algorithmic bias risks exacerbating healthcare disparities. Models trained on non-representative data (e.g., predominantly White cohorts) may underperform in minority populations (e.g., Black patients), potentially widening health inequities (48, 49). Mitigation requires multifaceted strategies: curating diverse datasets (e.g., via NIH “All of Us”), embedding fairness constraints during algorithm development, and rigorous subgroup performance validation.

### 4.4 Translational delays and collaborative intelligence

Translational delays further impede progress. Despite AI advances in cardiology and oncology separately, few models targeting cardio-oncology undergo prospective validation (49). Key barriers include limited clinical integration, workflow compatibility issues, and practitioner adoption challenges.

Addressing these translational barriers requires a fundamental reconceptualization of how clinicians and AI systems interact. Rather than positioning AI as an autonomous decision-maker that displaces human judgment, a more productive paradigm lies in collaborative intelligence—frameworks that deliberately orchestrate the complementary strengths of human expertise and machine computation (50). Recent evidence underscores the superiority of this approach: hybrid human–AI collectives have been shown to outperform individual physicians, standalone AI models, and groups composed solely of either humans or AI in complex diagnostic tasks, leveraging the distinct and complementary error profiles of each component to achieve higher aggregate accuracy (51).

In the cardio-oncology context, collaborative AI systems could manifest through several concrete mechanisms. First, interactive decision-support platforms can engage clinicians in multi-step, iterative dialogues where AI-generated recommendations are accompanied by explicit uncertainty quantification and are subject to clinician refinement (50). Such systems would not merely output risk scores but would actively solicit clinical context, patient preferences, and nuanced observations that escape structured data capture. Second, workflow-embedded AI assistants—designed through participatory co-design with end-users—can address compatibility issues by functioning within existing electronic health record (EHR) infrastructures and aligning with established clinical pathways rather than demanding disruptive workflow reconfigurations (51). Third, prospective validation frameworks that incorporate clinician feedback loops during model development and deployment can ensure that AI tools remain responsive to evolving clinical realities and user needs.

For clinicians to position themselves effectively in this AI-augmented future, several proactive strategies are warranted. Clinicians should cultivate foundational AI literacy—not necessarily to develop algorithms, but to critically appraise AI outputs, recognize potential failure modes, and communicate AI-derived insights to patients with appropriate nuance. Furthermore, clinicians must advocate for and participate in the design of AI systems that prioritize interpretability, fairness, and workflow compatibility from inception rather than as afterthoughts. Finally, clinicians should embrace their irreplaceable role in the collaborative intelligence ecosystem: providing the contextual reasoning, ethical judgment, and empathic communication that remain beyond the reach of computational systems. By actively shaping how AI is integrated—rather than passively receiving its outputs—clinicians can ensure that these technologies augment rather than undermine the humanistic foundations of cardio-oncology care.

Future success demands paradigm shifts toward collaborative innovation. Research should prioritize causal inference AI models that identify actionable biological mechanisms over correlative analyses. The ultimate goal is shifting from reactive toxicity management to proactive “toxicity-by-design” prevention—preempting cardiotoxicity through optimized treatment. Cross-disciplinary collaboration remains essential to build patient-centric, equitable AI systems that fulfill cardio-oncology's promise.

## 5 Conclusion and future directions

Artificial intelligence is fundamentally reshaping cardio-oncology, demonstrating unparalleled capacity to decode pathological mechanisms, predict cardiotoxicity with high accuracy, and personalize therapeutic decisions (Table 1). Yet this transformative potential remains nascent. Clinical translation necessitates dismantling barriers between oncology, cardiology, and computational science.

**Table 1:** AI research and applications in cardio-oncology.

Application Scenarios	Technology	Case Studies	Objectives	Results
Mechanism exploration	Organoid-based dynamic analyses	(19)	Revealed novel cross-organ pathogenic communication between breast cancer and the heart through the exosomal miR-216a-5p-mediated ITCH/TXNIP/NLRP3 pathway	
Cancer screening	CNN	Mia 2.0, Kheiron Medical Technologie s (52)	Prospective validation of AI safety and efficacy in real clinical workflows, improving early detection and optimizing efficiency	Screening performance +7% detection rate; additional recall rate only 0.16–0.30%, but positive predictive value (PPV) improved by 0.1–1.9%
Drug development	Utilizing molecular structure dynamics to generate risk classifications	DMFGA M (53)	Identifying potentially cardiotoxic molecules early in drug development	Accuracy rate: 81.7%
Image automation	CNN ultrasound analysis	EchoNet-Dynamic (2020 ) (36)	Improve detection accuracy and reduce errors	EF prediction error was only 4.1% (compared to 13.9% for manual error), with a heart failure classification accuracy rate of 97% (AUC 0.97).
Risk stratification	Multimodal integration of cardiac ultrasound images, cardiac function indicators, and clinical data	(37)	Identify high-risk patients and reveal key risk factors	CTRCD prediction sensitivity 88%, AUC 0.83
Early warning	AI-ECG/GLS	(38, 54)	Replacing expensive ultrasound/MRI examinations with standard electrocardiograms/GLS + AI to achieve widespread cardiac safety monitoring for cancer patients	RUC = 0.86 (17)
Outcome assessment	image segmentation	DenseNet-121 CN N (21)	Objectively evaluate ultrasound image quality, improve LVGLS reliability, and reduce false positive rates	Quantify image quality and strain reliability. CTRCD false positive rate reduction.

To fully harness AI as a cardiovascular guardian for cancer survivors, we propose three critical actions: First, establish dedicated infrastructure like “AI-HOPE” (55) in cardio-oncology field—integrated platforms harmonizing cancer-agnostic EHRs, multi-omics, and wearable data. Such repositories must proactively include underrepresented groups (elderly women, ethnic minorities) to correct existing diagnostic biases. Second, pioneer pragmatic AI-guided clinical trials, including randomized studies comparing AI-ECG monitoring against standard care in high-risk regimens, and adaptive trials validating digital twin-driven therapeutic personalization. Third, innovate regulatory frameworks requiring continuous real-world performance monitoring rather than static evaluations.

The field's future lies not merely in incremental predictive improvements but in engineering cardiotoxicity prevention. Through causal AI, federated learning, and digital twins, we can transition from passive damage control to proactive safety-by-design paradigms—ensuring cancer survivors’ longevity remains uncompromised by preventable cardiovascular complications. Realizing this vision demands immediate, concerted collaboration across academia, industry, and regulators. With technical tools now available, patients urgently await their clinical translation.
